# Vascular complications in craniopharyngioma-resected paediatric patients: a single-center experience

**DOI:** 10.3389/fendo.2024.1292025

**Published:** 2024-04-11

**Authors:** Barbara Castelli, Mirko Scagnet, Federico Mussa, Lorenzo Genitori, Iacopo Sardi, Stefano Stagi

**Affiliations:** ^1^ Department of Health Sciences, University of Florence, Florence, Italy; ^2^ Neuro-oncology Department, Meyer Children’s Hospital IRCCS, Florence, Italy; ^3^ Neurosurgery Department, Meyer Children’s Hospital IRCCS, Florence, Italy; ^4^ Struttura Organizzativa Complessa (SOC) Diabetology and Endocrinology, Meyer Children’s Hospital IRCCS, Florence, Italy

**Keywords:** paediatric neuro-oncology, craniopharyngioma, deep venous thrombosis, vascular complications, neurosurgery

## Abstract

**Background:**

Craniopharyngioma (CP), although slow growing and histologically benign, has high morbidity, mostly related to hypothalamus-pituitary dysfunction and electrolyte imbalance. Increased risk of vascular complications has been described. However, data are still poor, especially in the paediatric population. The aim of our study was to evaluate the occurrence, timing, and predisposing factors of deep venous thrombosis (DVT) and other vascular alterations in neurosurgical paediatric CP patients.

**Materials and Methods:**

In a single-centre, retrospective study, we investigated 19 CP patients (11 males, 8 females, mean age 10.5 ± 4.3 years), who underwent neurosurgery between December 2016 and August 2022, referred to Meyer Children’s Hospital IRCCS in Florence.

**Results:**

Five patients (26.3%) presented vascular events, which all occurred in connection with sodium imbalances. Three DVT (two with associated pulmonary embolism, in one case leading to death) developed in the post-operative period, most frequently at 7-10 days. Elevated D-dimers, a reduced partial activated thrombin time and a prolonged C-reactive protein increase were highly related to thrombotic vascular events. One case of posterior cerebral artery pseudoaneurysm was described soon after neurosurgery, requiring vascular stenting. Superficial vein thrombophlebitis was a late complication in one patient with other predisposing factors.

**Conclusion:**

CP patients undergoing neurosurgery are at risk of developing DVT and vascular alterations, thus careful follow-up is mandatory. In our study, we found that the phase of transition from central diabetes insipidus to a syndrome of inappropriate antidiuretic hormone secretion may be a period of significant risk for DVT occurrence. Careful vascular follow-up is mandatory in CP-operated patients.

## 1 Introduction

Craniopharyngioma (CP) is a slow growing intracranial neoplasm, arising from remnants of Rathke’s pouch (1) and representing about 6–9% of paediatric brain tumours (2). Although histologically benign, the disease has considerable morbidity and mortality mostly due to hypothalamus-pituitary dysfunction (3).

Treatment strategies in CP vary from radical to conservative surgery and radio-oncological approaches (3). The anatomical location of CP is key for selecting the appropriate surgical technique (4), which includes transcranial approaches (TCA) or a paediatric endoscopic endonasal approach (EEA) (2). The most common postoperative complications of CP are pituitary-target gland axis dysfunction and electrolyte disorders (5). Fluid-electrolyte disturbances should be carefully managed also with adequate replacement of central hypothyroidism and hypocortisolism, to avoid permanent neurological sequelae (3).

Central diabetes insipidus (CDI), syndrome of inappropriate antidiuretic hormone (ADH) secretion (SIADH), cerebral salt wasting (CSW) disease and adipsic diabetes insipidus (ADI) can occur individually or exist simultaneously making diagnosis and management extremely challenging (6). CDI is already present in 16–55% of patients before surgery, and in up to 90% after surgery (3). A thorough understanding of the fluid-electrolyte phase which the patient is going through is essential for the optimal management of these disorders (3).

Post-surgical hormonal and electrolyte complications are worrying not only in themselves, but may potentially represent risk factors for other conditions, such as thrombotic events. It is known that tumours are an important cause of thrombosis in children (7). Risk factors for developing cancer-related thrombosis include patient, disease, or treatment-related influences (8). Most paediatric data on venous thrombotic events (VTE) focus on patients with haematological cancers or other solid tumours. There is limited data for patients with central nervous system (CNS) tumours (8).

The aim of our study was to describe the occurrence, timing, and predisposing factors of VTE and other vascular events in CP-operated paediatric patients, in the post-surgical phase and in the follow-up period.

## 2 Materials and methods

This was a single-centre, retrospective study. We investigated 19 patients diagnosed with CP, who underwent neurosurgery between December 2016 and August 2022, referred to Meyer Children’s Hospital IRCCS in Florence. We assessed the risk for developing post-operative thrombosis, describing patients’ history, searching for causative factors, and evaluating fluid electrolyte profiles at the time of the complication. We also assessed the risk for other early and late vascular events in the same cohort.

This study was performed in accordance with the principles of the Declaration of Helsinki and approved on 14th September 2020 by the Meyer Children’s Hospital Ethics Committee (project number 256/2022). Written informed consent was obtained from enrolled children’s parents.

Inclusion criteria were patients aged < 18 years, who underwent CP surgery.

Exclusion criteria were: (1) patients with a prior history of DVT and/or pulmonary embolism (PE); (2) patients who received anticoagulant or antiplatelet drugs before surgery; and (3) already known coagulation dysfunction caused by blood system diseases.

Included patient’s preoperative clinical indices, laboratory results, and operative records were retrospectively reviewed. The parameters of the analysis involved basic information of patients (age, sex, height, weight, and body mass index BMI), blood test results after operation and at the time of vascular event, surgical data (type, radicality and duration of surgery). Histological diagnosis was based on the World Health Organization classification (9, 10). Enrolled patients were observed during hospital stays and during a follow-up period. Timing of postoperative ultrasonography was determined by attending neurosurgeons, based on patients’ symptoms and physical examinations suggestive of DVT or massive increase of postoperative D-dimer. The diagnosis of DVT was made according to the guidelines (11, 12).

All patients were preventively treated with compression stockings and intermittent pneumatic compression devices after operation. Patients did not routinely receive postoperative anticoagulant treatment. Following the ultrasonographic detection of a thrombus, patients were required to lift their limbs and received subcutaneous injected low-molecular-weight heparin (LMWH) (12). In CP-operated patients we reported also vascular alterations other than thrombotic.

Descriptive statistics were used. Comparison of categorical variables was conducted using the chi square test or Fisher’s Exact test if there was a small (< 5) expected cell size. All statistical tests were two-tailed and a p < 0.05 was considered statistically significant.

## 3 Patients’ characteristics

Demographic and clinical characteristics were recorded for each participant and included sex and surgical approach (craniotomy vs. no craniotomy, partial or total resection). Patients who received one or two craniotomies, or multiple approaches that included a craniotomy were categorized in the craniotomy group. Patients who received an endoscopic resection, ommaya-closed, ommaya-open, a transsphenoidal, were categorized in the no craniotomy group. In the follow-up some patients relapsed needing further approaches.

## 4 Anthropometrics

All patients underwent a physical examination and auxological evaluation. We recorded height, weight and BMI (13). BMI was calculated by dividing the patient’s weight in kilograms by the square of height in meters (14). Height and BMI were normalized for chronological age by conversion to SD scores (13). Pubertal development was classified according to the Marshall and Tanner criteria (15). Age-related reference values for height were those currently used in Italy (13). Sex- and age-adjusted BMI z-scores were calculated using the 2000 Centre of Disease Control (CDC) growth charts.

## 5 Results

We retrospectively investigated a total of 19 CP patients referred to our tertiary care centre (11 males, 8 females, mean age 10.5 ± 4.3 years) who met the inclusion criteria. The median age at enrolment was 10.8 years (range 3-17 years).

DVT episodes following neurosurgery were detected in 3 patients (2 females and one male, 15.8%; p < 0.05, mean age 10.0 ± 4.4 years, median age 8 years, all prepubertal patients). PE occurred in two of them, causing one patient’s death (a female, 5.3%).

Through the contrastive analysis of categorical variables between patients with and without DVT, we found that demographical data (age, BMI, sex) and surgical data (type, radicality and duration) were not significantly associated with DVT. We found that D-dimers and a reduced partial activated thrombin time after surgery were highly related to vascular events, whereas other coagulation indicators (i.e., prothrombin activity, INR, and fibrinogen) were not related. All the patients with post-operative vascular issues, including patient 15 who developed pseudo aneurysm of the right posterior cerebral artery (PCAP), presented elevated D-dimers (> 1 μg/ml) at the time of vascular complication. D-dimers were checked in the majority of patients who did not develop early post-surgical vascular events (range 0.2-0.6 μg/ml).

In patients with thrombotic vascular complications on antithrombotic treatment, at first reassessment (at 1-14 days depending on clinic), the patient, who died of PE, presented an exponential massive increase, patient 14 showed stable values, patient 15 had progressively decreasing values.

Other factors which were not significantly associated included platelets, leukocytes, blood creatinine and thyroid function.

Interestingly 10/19 of patients (52.6%) presented a total calcium level ≤ 2.12 mmol/l in the postoperative period: one with severe hypocalcaemia developed massive DVT. When pre-operatively calcium level was assessed, it resulted normal, so surgery surely contributed to the development of hypocalcaemia. Vitamin D was not checked in this phase, but during follow-up and it was usually insufficient.

As known, hypercholesterolemia and hypertriglyceridemia are predisposing factors for thrombotic occurrence.

Sodium imbalance and prolonged increased C-reactive protein without signs of infections were significantly associated with the development of thrombosis.

All patients, who presented a vascular event, also presented a sodium concentration outside the normal range and all affected patients had post-surgical CDI causing electrolyte imbalance.

**Figure 1:**
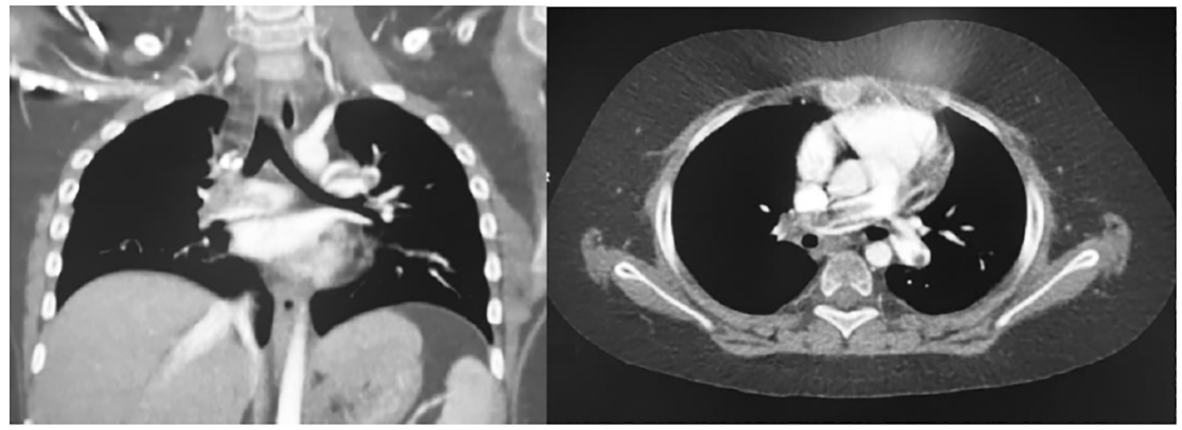
Coronal and axial chest CT images of Pulmonary embolism in patient 12.

**Figure 2:**
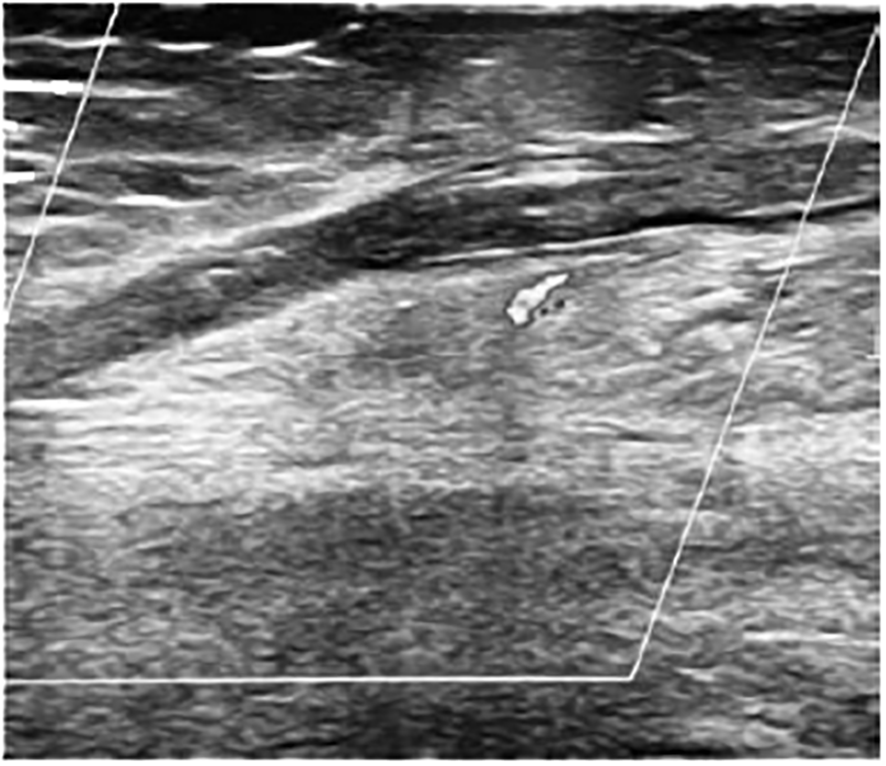
Utrasound image of femoral thrombosis in patient 18.

Three patients (two carriers of a central venous catheter and one obese) developed vascular thrombosis, usually at 8-10 days after surgery during the transition from CDI to SIADH. One female patient died of massive PE (patient 12, **Figure 1**), one female patient presented a massive central thrombotic apposition from the right femoral central venous catheter to the inferior vena cava (IVC) (patient 17), and another male patient had a venous thrombosis of the popliteal and distal femoral vein (**Figure 2**), with subsequent PE (patient 18). At the time of vascular complication, patient 12 was in a persistent hypernatremic post-surgical CDI phase just before reverting to SIADH phase, patient 17 was frankly at the beginning of SIADH phase. Patient 18 presented the massive thrombosis in the post-surgical permanent CDI phase which follows the SIADH phase.

In patient 17 initial recanalization was found at the doppler evaluation after 7 days, in patient 18 after 2 days.

Two days after surgery, while in a hyponatraemic state with a subarachnoid haemorrhage requiring intensive care, one 15-year-old male presented an acceleration in flows on transcranial doppler studies, thus intra arteria nimodipine was injected. The presence of an acquired PCAP was found, for which stenting was performed.

Five months after neurosurgery, one 15-year-old female was admitted because of walking difficulties due to tumour recurrence. She presented bilateral vision reduction and important post-surgical weight gain. A doppler ultrasound revealed a thrombus extended along the entire length of the small saphenous vein, and thrombophlebitis (TP) was diagnosed, just a few days after the beginning of estrogenic replacement therapy. Her major predisposing factors were related to her previous neuro oncological history with the associated complications, such as the developed obesity.

Clinical and laboratory characteristics of patients with vascular complications at the time of the event are described in **Table 1**.

**Table 1:** Clinical and laboratory characteristics of patients with vascular complications at the time of vascular event.

		Patient number				
		12	14	15	17	18
Type of event		DVT^1^+PE^2^	TP^3^	PCAP^4^	DVT^1^	DVT^1^+PE^2^
Time after surgery	Days	8	154	2	10	34
Sodium [135 - 145]	mmol/L	**156**	**157**	**124**	**133**	**158**
Calcium [2,2 – 2,6]	mmol/L	**2.1**	NA^5^	**2.2**	2.3	**1.7**
D-dimers [< 0.50]	μg/ml	**9.7**	NA^5^	**1.3**	**2.8**	**2.5**
Fibrinogen [2.0 – 4.0]	g/L	3.0	**4.3**	3.4	3.7	**5.1**
Prothrombin time [70 - 100]	%	100	100	92	100	74
Activated partial thromboplastin time [26 - 40]	Seconds	**17**	**21**	33	**24**	**19**
Antithrombin III [66 - 124]	%	115	90	109	**129**	96
International normalized ratio [0.8 – 1.2]		0.9	NA^5^	NA^5^	1	1.2
White blood cells [3,5– 14]	x 10^9/L	**16.6**	4.8	14	**15.7**	8.0
Neutrophils [25-65]	%	59.7	49.9	**69.9**	**71.9**	47.1
Platelets [150-590]	x10^9/L	264	204	283	424	325
C reactive protein [<10]	mg/L	**23.2**	2.9	**22.6**	**21.1**	**22.4**
Creatinine [age-specific limits]	umol/L	28.2	73.4	31.8	31.0	52.2
Free T4 [10-26]	pmol/L	15.6	14.8	14.4	16.0	11.8
Cholesterol [<4.39]	mmol/L	NA^5^	4.01	NA^5^	NA^5^	**6.0**
Triglycerides [<2.33]	mmol/L	NA^5^	**2.6**	NA^5^	NA^5^	**2.7**
Body Mass Index (BMI) [18.5-22.9]	Kg/m2	21.81	**28.8**	21.0	**18.4**	**30.6**
BMI SDS WHO	SDS	**2.1**	**1.9**	0.4	1.4	**2.5**
Weight	Kg	33.0	62.0	52.0	25.0	59.8
Weight SDS	SDS	1.1	0.3	0.3	0.4	0.7
Height	Cm	123.0	146.8	157.5	116.5	139.9
Height SDS	SDS	-1.4	**-2.4**	-1.6	-1.2	**-4.1**
Age	years	8	16	15	7	15
Sex	M/F	F^6^	F^6^	M^7^	F^6^	M^7^
Central Venous Catheter carrier	YES/NO	yes	no	yes	yes	no
Other		no	ERT^8^	no	no	no

^1^DVT, deep venous thrombosis; ^2^PE, pulmonary embolism; ^3^TP, thrombophlebitis; ^4^PCAP, pseudo aneurysm of posterior cerebral artery; ^5^NA, not applicable; ^6^F, female; ^7^M, male; ^8^ERT, oestrogen replacement therapy.

Not-standard values in bold.

## 6 Discussion

In CP-resected patients, vascular complications, especially thrombotic events, must be early recognized. In the adult population, Qiao et al. noticed that the overall incidence of venous thromboembolism after surgical resection of major sellar region tumours was clinically significant, especially in patients with CP (16). Recently, Liu et al. also reported a high incidence of postoperative thrombosis-related complications in patients with craniocerebral malignant tumours undergoing resection (17). In the paediatric population, Wang et al. reported that longer operation time was the main primary cause of thrombosis in children with intracranial diseases (7). However, the literature is still scarce, and few data exist about the occurrence, the association with fluid electrolyte status and the possible prevention of this phenomenon. Our study suggests that a strict follow-up with attention to vascular complications is mandatory in CP operated paediatric patients. In 2022, Lambert et al. conducted a systemic review of the literature showing that venous thromboembolism appears to have a higher incidence among patients with CDI, and recommended a more aggressive surveillance in paediatric neurosurgical patients with CDI (18). Our study confirms that DVT is a frequent occurrence in the postsurgical phase of CP. In particular, the period of transition to the SIADH phase may represent a significant risk period for DVT in these patients, therefore in this phase ultrasound screening and D-dimers monitoring may be useful.

The course of postoperative fluid-electrolyte disturbances usually has a triphasic pattern. The first phase, lasting approximately 1–7 days, is characterized by ADH deficiency, resulting from neuron dysfunction (3) and requiring appropriate fluid replacement (3). It is followed by SIADH, occurring from 2 to 14 days (3), due to unregulated release of ADH from recovery of secreting neurons (6) and causing oliguria and hyponatremia (3), managed with fluid restriction and hypertonic saline when necessary (3). The third phase consists of permanent CDI from the death of ADH secreting neurons (6) and requiring long-term desmopressin (dDAVP) replacement (3). Hyponatremia may be aggravated by CSW that triggers inappropriate ADH release (3): it usually occurs within the first 10 days after the neurosurgical procedure (3), and causes dehydration (3).

In addition to primary pituitary and electrolyte dysfunction, weight can increase after CP management due to pituitary dysfunction, steroid replacement and hypothalamic injury (2). In a study Yaxian et al. found that hypothalamus obesity occurred in 14.58% of the analysed patients (5). In our study, a patient, who developed a late vascular complication, had a severe post-surgical obesity. It is known that hypothalamic obesity, compounded by a disruption of the hypothalamic pituitary axis, increases the risk of vascular sequelae (19).

It is known that in children tumours are important causes of thrombosis (7). However Howie et al. demonstrated that paediatric patients with CNS tumours experience a significantly lower incidence of VTE compared with patients with non-CNS cancer, despite undergoing similar treatment (8). The blood-brain barrier (BBB) could have a role in limiting treatment and disease-related systemic inflammation (8). Instead, children operated on sellar and suprasellar tumours have an increased risk of developing venous thrombosis (20). Vessel wall injury, hypercoagulability, and abnormal venous flow are established factors contributing to thrombosis (21). Moreover, the brain intrinsically contains high expression levels of tissue factor, which is a well-recognized procoagulant substance (22). Brain tumour resection by craniotomy is associated with a high risk of DVT (23): it may result in the release of coagulants into the blood leading to systemic hypercoagulability and consequent development of DVT in the lower extremities after surgery (24). In our cohort one patient, who developed DVT, had been subjected to craniotomy. The occurrence of vascular thrombosis in CP-affected patients has already been shown. Furtado et al. reported two cases of IVC thrombosis following CP surgery (20). A case of CP with intra tumoral bleeding coexisting with chronic cerebral venous sinuses thrombosis has also been described (1). However, the risk of venous thromboembolism has not been extensively assessed in paediatric CP patients undergoing brain surgery (25). In a study performed by Wang et al. DVT was most frequently associated with operated CP in respect to other cranial tumours, suggesting that the increased vascular risk might also be related to the possible effect of the post-surgical hormone replacement treatment (7), which intrinsically characterizes this type of tumour. The same group reported that thrombosis occurred 10 days after surgery, suggesting that DVT screening should be introduced 10 days after operation (7). This time frame (7) correlates to the SIADH phase that typically follows surgery, confirming our data that show this period as one of the main risk factors in the development of DVT. In our cohort, DVT episodes following neurosurgery were detected in 3 patients (15.8%) and in one patient the thrombotic event was fatal. Ultrasound Doppler is currently the most widespread technique for detection of DVT (26). We must consider that in our study ultrasound examination was performed only for patients with suspected DVT, which may have led to a slightly lower incidence of diagnosed thrombosis. A D-dimer post-surgical monitoring might also be useful. A number of pathological mechanisms have been postulated as the basis of post-surgical CP vascular complications, including acute and chronic vascular changes due to vasoactive peptides and hormones from the hypothalamus, disturbances of microcirculation as a consequence of fluid-electrolyte imbalance and hypovolemia, and spasm of basal arteries due to subarachnoid blood (27). Kamal et al. assessed on rats the direct effect of CP fluid on the femoral vessels, showing that it induces vasospasm from day 4 of the instillation onwards (27). Both inadequate dDAVP replacement and dDAVP therapy have been associated with thrombosis (28). Indeed, desmopressin therapy transiently increases factor VIII coagulation activity and von Willebrand factor by releasing these elements from endothelial cells into the plasma, leading to an increased risk of thrombosis (20, 28). All our DVT-affected patients had post-surgical CDI, requiring desmopressin replacement. Hormonal changes, such as decreased cortisol, hypothyroidism, and altered water and electrolyte balance, should be corrected before surgery (29). In the immediate postoperative period desmopressin was administered only as necessary, to ensure a correct assessment of electrolytes. In fact in the post-operative period dDAVP replacement dose should be titrated with caution because its long half-life during this phase may complicate the subsequent hyponatraemic phase of the triphasic response: regular use of dDAVP should only be done when CDI is persistent and permanent (3). Stress doses of corticosteroids were given to cover the stress of surgery and in the post-operative period until the assessment of hypothalamic-pituitary-adrenal axis (3, 29). Dexamethasone is a standard treatment after brain tumour surgery for treating peritumoral edema and transient neurologic sequelae of brain retraction/manipulation after surgery (30). Nevertheless, sparse evidence exists in the literature regarding postoperative steroid dosing to guide clinicians, even if the reduction of steroid doses to the lowest effective dose is a mandatory for improving the quality and safety of care. In Meyer Children’s Hospital IRCCS there is a longstanding practice of treating patients with brain tumour with high-dose dexamethasone tapered in the immediate postoperative setting (31). Hydrocortisone stress therapy (50–100 mg/m2, then 50–100 mg/m2 every 24 hours) must be given intravenously before and during brain surgery and resumed postoperatively in place of dexamethasone at a dose of 10 mg/m2 orally, depending on a patient’s general condition and pain control, and possibly stopped once the normal function of the hypothalamic–pituitary–adrenal axis is confirmed.

The development of fusiform intracranial aneurysm after surgical removal of CP is another well-known phenomenon in the surgical approach to CP (32–34), mostly related to the extremely challenging location of the tumour. Fusiform dilatations could be caused by a focal arterial wall weakening after dissection of the tumour or might be a consequence of injury to the vasa vasorum, being more common in centres that aim for total resection (33). In our cohort, one patient (5.3%) presented the condition and was consequently admitted to intensive care. The development of aneurysms and radiation-induced vasculopathy must be borne in mind during the assessment of CP patients who have undergone postoperative radiotherapy (32). Paediatric patients with CP represent a particularly vulnerable group: several factors contribute to increase the risk of vascular complications (34).

Radiation has been shown to decrease the recurrence rate of CP to 21% (34), even if it is known that the treatment causes neurocognitive and neuropsychological impairments (34, 35). Radiation can also induce vascular injuries, such as degeneration of internal elastic tissue, endothelial damage, and degradation of the vascular wall (36). Radiation-induced large vessel cerebral vasculopathy has been reported in 6.7% of patients with a median time to development of 1.5 years (37). Other vascular complications and the development of pseudoaneurysms and aneurysms have been described several years after brain irradiation, even if aneurysm development seems less common compared with vascular stenosis development after radiation therapy (38). In our cohort, in relapsed CP patients subjected to radiation treatment, vascular events did not occur in the follow-up period.

Hypocalcaemia, common in CP patients in the post-operative period, is also common in shocked trauma patients (39). Our study confirms that low calcium may be associated with ischemic cell death (40) due to neurosurgery, and disruption of adherents junctions between endothelial cells (41). It is known that hypocalcaemia is a marker for the depth of brain damage as a result of various pathological mechanisms (42). However, the literature is still scarce in this field and further data are needed. Previous studies have also identified hypocalcaemia as an independent predictor of mortality after acute pulmonary thromboembolism (43).

In conclusion, in our cohort, considering both the post-surgical period and the follow-up up phase, 5 of the analysed patients developed vascular complications (in one PE was fatal). Compared to previous studies, we found that the incidence of DVT was 15.8%, greater than previously reported (8). Our study suggests that thrombotic complications should always be carefully monitored after CP neurosurgery and screening by doppler ultrasound should be considered in patients with more consistent risk factors, especially in those with important electrolyte imbalances during the transition to the SIADH phase. We found that D-dimers (> 1 μg/ml), a short aPTT (< 26 s), a prolonged C-reactive protein increase (> 10 mg/L) after surgery were highly related to thrombotic vascular events. In brain tumour resection by craniotomy Shi et al. reported a higher risk of DVT, related to older age, BMI, preoperative APTT, D-dimer, tumour histology, and surgery duration (23). Differently, we did not find an association with BMI and duration of surgery. At the time of post-surgical vascular complications, D-dimers value shot up, therefore the inclusion of D-dimers in the general postoperative coagulation check-up could be considered since this value could be strongly predictive of increased risk and incipient vascular complication. It is known that high D-dimer values cannot be used for diagnosis of paediatric VTE, as there are no clinical studies confirming their effectiveness in the paediatric population (11, 44). Moreover, in children D-dimer levels showed better discriminative and predictive ability for DVT in an exploratory sample of patients with no underlying conditions or co-morbidities at the time of diagnosis (45). However, our experience shows that in such complex situations, it could be extremely useful for guidance, considering also that it should be checked in routinely post-surgical haematological re-assessment, already frequently performed for the hormonal electrolyte imbalance risk. Anticoagulant treatment was tailored to each individual needs and referred on the paediatric consensus-based guidelines (46). The potential role of serial D-dimers to predict recurrence needs to be determined (12).

In our study, we found that the phase of transition from central diabetes insipidus to a syndrome of inappropriate antidiuretic hormone secretion may be a period of significant risk for DVT occurrence. Careful vascular follow-up is mandatory in CP-operated patients. We think that this study provides some useful insights into the timing and probability of the postoperatively occurrence of DVT in post-operation CP patients. However, it also has several limitations, such a single-centre design and the lack of deep vein ultrasound examination following operation in the entire CP operated population, which may have led to a slightly lower incidence of thrombosis. Careful vascular follow-up is mandatory in CP-operated patients.

## Data availability statement

The raw data supporting the conclusions of this article will be made available by the authors, without undue reservation.

## Ethics statement

The studies involving humans were approved by Ethics Committee of Anna Meyer Children’s Hospital of Florence. The studies were conducted in accordance with the local legislation and institutional requirements. Written informed consent for participation in this study was provided by the participants’ legal guardians/next of kin. Written informed consent was obtained from the minor(s)’ legal guardian/next of kin for the publication of any potentially identifiable images or data included in this article.

## Author contributions

BC: Writing – original draft, Methodology, Formal analysis, Data curation, Conceptualization. MS: Writing – review & editing, Visualization, Validation, Supervision, Resources. FM: Writing – review & editing, Visualization, Validation, Supervision. LG: Writing – review & editing, Visualization, Validation, Supervision. IS: Writing – review & editing, Visualization, Validation, Supervision. SS: Writing – original draft, Writing – review & editing, Visualization, Validation, Supervision, Data curation, Conceptualization.
